# Corneal Perforation Caused by Eyelid Margin Trichilemmal Carcinoma: A Case Report and Review of Literature

**DOI:** 10.3389/fmed.2022.896393

**Published:** 2022-05-13

**Authors:** Liying Zhang, Zhirong Lin, Huping Wu, Shangkun Ou

**Affiliations:** ^1^Eye Institute and Affiliated Xiamen Eye Center of Xiamen University, School of Medicine, Xiamen University, Xiamen, China; ^2^Department of Ophthalmology, The Affiliated Hospital of Guizhou Medical University, Guiyang, China; ^3^Fujian Key Laboratory of Ocular Surface and Corneal Diseases, Xiamen University, Xiamen, China

**Keywords:** eyelid margin trichilemmal carcinoma, blepharokeratoconjunctivitis, corneal perforation, case report, review (article)

## Abstract

**Background:**

Trichilemmal carcinoma (TLC) is a rare malignant adnexal tumor most commonly found in the elderly, usually affecting the scalp, eyelids, neck and face. Here, we first reported a rare case of corneal perforation caused by eyelid margin TLC.

**Case Presentation:**

A 68-year-old female presented with 2 months history of unprovoked redness, pain and blurred vision in the left eye. On slit-lamp examination, a 1 × 2 mm sized aseptic corneal perforation embedded by iris prolapsed was noted. Upon detailed case investigation, we speculated that the severe meibomian gland dysfunction (MGD) and subsequent Blepharokeratoconjunctivitis (BKC) could have led to corneal perforation. The patient underwent penetrating keratoplasty to prevent ulcer enlargement and infection. However, several tiny nodules gradually developed on the eyelid margin postoperatively, accompaniedby with bleeding, burst and madarosis postoperatiely. Subsequently, biopsy revealed the growth of TLC on the eyelid margin, and lesionectomy was immediately conducted During the 1-year follow-up period, no local recurrence or metastasis was observed.

**Conclusions:**

To date, there has not been any report of corneal perforation caused by eyelid margin TLC. Consideration of the clinical presentation, feature and histopathologist will be benefit for the dignoses and treatment of TLC. Ensuring a smooth eyelid margin by total excision of TLC and consistent followup of patient will avoid recurrence.

## Background

Trichilemmal carcinoma (TLC) is a rare malignant adnexal tumor oriented fom the external hair sheath. Usually, it develops as a large, solitary, multilobular lesion (1). The TLC performs a typical histopathology of trichilemmal keratinization and a peripheral palisading pattern that indicats the cells from immature pilosebaceous units. The cells are classical large, polygonal, clear cells with eccentric nuclei and atypical mitosis. Cells usually exihibit high-proliferation acticity, Ber-EP4 negative and lack hair follicle differentiation (1). It is a locally aggressive tumor that predominantly affects the scalp, eyelids, neck and face of the elderly (1–6). The tumer can be complete excision and there is no evidence of recurrence after operation (7, 8). There were some case reports about the eyelid disorder associated with TLC (5, 9), while the involvement of eyelid margin by TLC and subsequent blepharokeratoconjunctivitis (BKC) have not been reported yet. Here, we report the case of corneal perforation caused by a TLC at eyelid margin.

## Case Presentation

**Figure 1:**
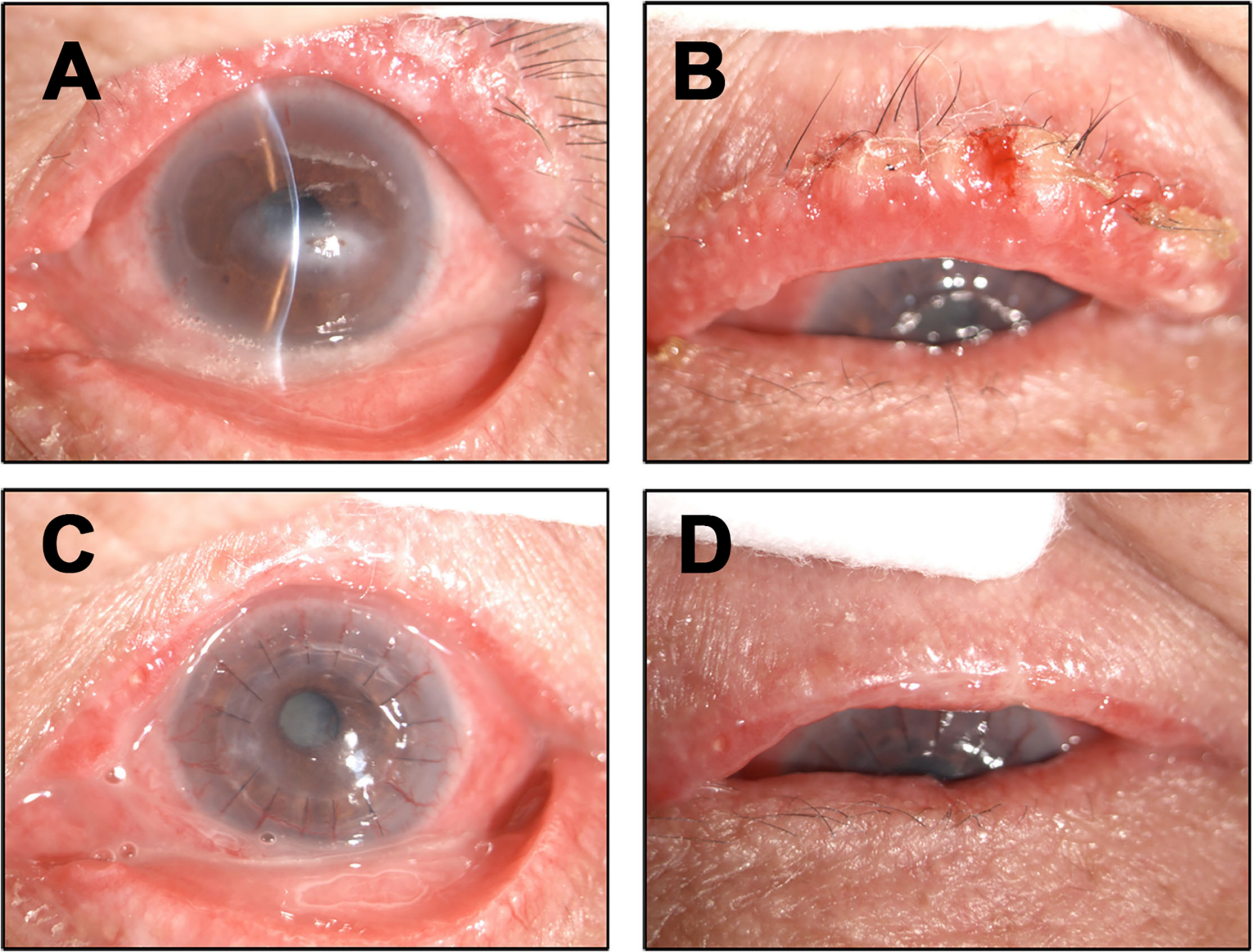
Ocular surface findings. **(A)** Clinical photographs at the first visit. The left upper eyelid had slight keratinization, several aberrant lashes with clogged meibomian gland, conjunctival was congestions corneal epithelial punctate defect, perforation. **(B)** During the second month review after penetrating keratoplasty the patient presenting with small nodules accompanied with bleeding, burst and madarosis in the left eyelid margin. **(C,D)** During 1 year followup the corneal graft was transparent and the eyelid margin did not show any TLC recurrence and metastasis.

**Figure 2:**
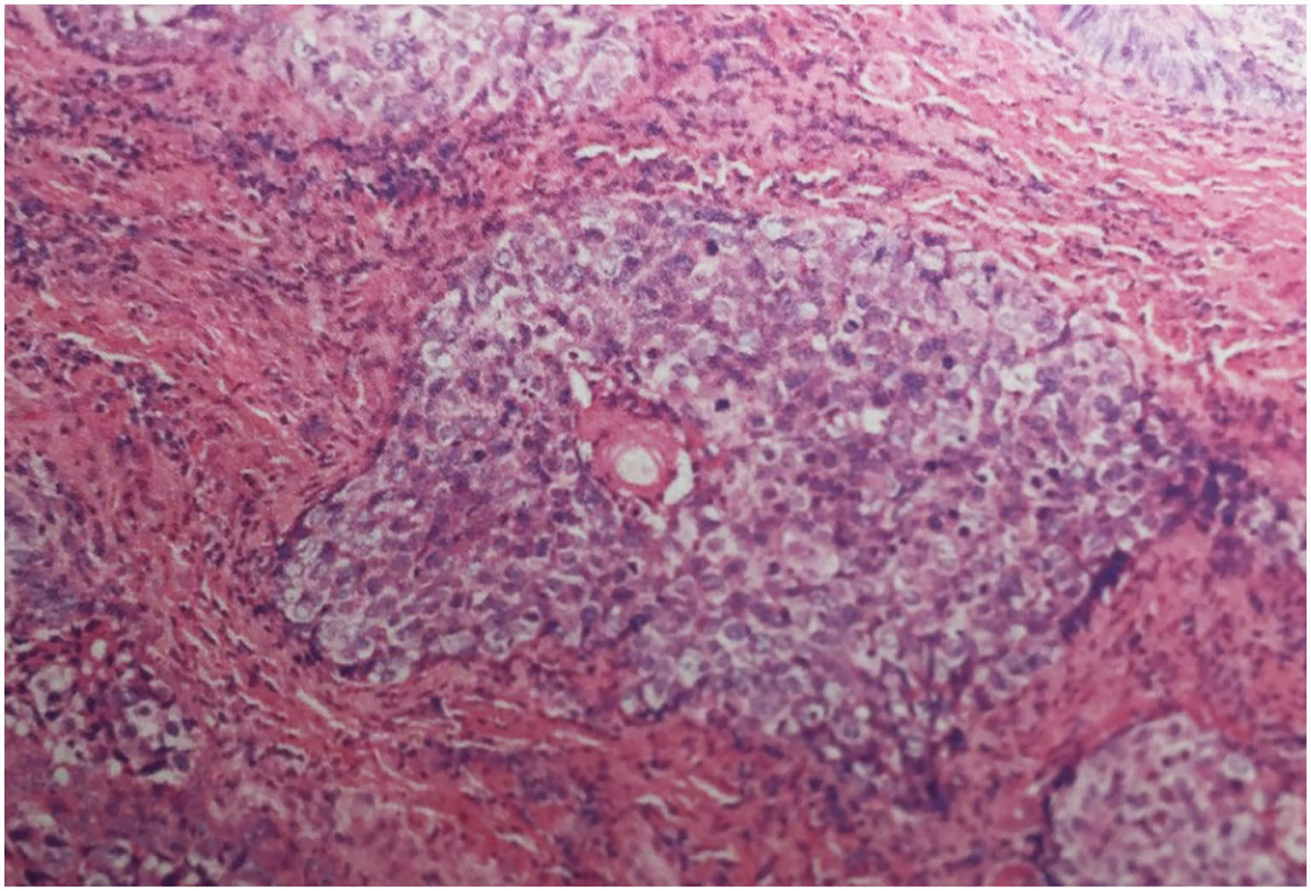
Histological section demonstrating infiltrative lobules of clear cells composed of large, polygonal, clear cells with eccentric nuclei (Haematoxylin-eosin stain; magnification ×200).

A 68-year-old female presented with 2 months history of redness, pain and blurred vision in the left eye. The slit-lamp examination revealed slight keratinization and multiple abnormal lashes on the left upper eyelid, clogged meibomian glands, and congestion and edema of the conjunctiva. Furthermore, diffused corneal epithelial punctate defect and neovascularization, especially a 4 × 3 mm sized irregular corneal epithelial defect was observed at the temporal cornea. The most crucial observation was the presence of 1 × 2 mm sized aseptic corneal perforation existed in the central cornea, causing iris incarceration (Figure 1A). The patients did not have a history of eye surgery or ocular trauma, or the immune-related disorders of rheumatoid arthritis, sjögren's syndrome, and infectious keratitis typically associated with corneal perforation. Routine investigations were also performed to rule out mite infestation, bacterial or viral infection, allergic blepharositis and seborrheic blepharositis. Accordingly, we predicted that the rough eyelid margin resulting from severe MGD might be responsible for persistent corneal epithelial defect, neovascularization, perforation, and blepharitis. In order to prevent ulcer enlargement and intraocular infection, penetrating keratoplasty with a 7.5 mm graft size was performed immediately. During 2 months follow-up period, the graft remained clear and no serious complications occurred. However, several small nodules gradually developed on the rough eyelid margin which accompanied with bleeding, burst and madarosis (Figure 1B). Subsquently, to probe the reason of persistent rough eyelid we performed biopsy. Suprisingly, we observed a tumor attached to epidermis which infiltrated the hair follicle within the dermis. We diagnosed the tumor as TLC, as the sections demonstrated classical large, polygonal, clear cells with eccentric nuclei and trichilemmal keratinization (Figure 2). TLC was immediately managed by lesionectomy. Postsurgery, the patient underwent the routine treatment for the ocular surface and was transferred to the oncology department for further general examination, which revealed no metastasis, so that chemoradiothreapy was not performed. During 1 year follow-up period, there was no evidence of local recurrence and metastasis, and the corneal graft was transparent (Figures 1C,D).

## Discussion and Conclusions

In 1976, Headington et al. (10) firstly described TLC as “a histologically invasive, cytologically atypical, clear cell neoplasm of adnexal keratinocytes which is in continuity with the epidermis and/or follicular epithelium”. It is commonly seen on the sun-exposed skin of the elderly (9, 11, 12), especially affecting the age group of 40-year-old or older (13), without gender pre-dilection (11). Dailey et al. (9) was the first to report TLC growth in the eyelid margin, however, the involvement of the eyelid margin and the subsequent corneal perforation is rarely reported and the pathogenesis of TLC is not known clearly. Previous studies have postulated that the actinic damage (14), transformation from benign trichilemmoma (7) or long term low dose irradiation (15) could be the etiology of TLC. Histologically, TLC is charecterised by single, exophytic nodular appearance, measuring less than 2 cm in diameter, and sometimes it complicates with ulcers and keratosis (7, 10, 15). Microscopically, TLC usually demonstrates proliferative lobules centrered on pilosebaceous, composed of polygonal clear cells with high-grade mitotic potential (7, 12, 13). But in our case we noted several diffused nodules, ulcerations on the upper eyelid margin, and was poorly circumscribed. There was no change in the palpebral conjunctiva.

TLC are often confused with other skin cancer, such as clear cell basal cell carcinoma (BCC) with few keratin cysts and peripheral palisading cells within the basaloid islands, squamous cell carcinoma (SCC) with clear cell differentiation of flat cells, trichoepithelioma which are composed of variety of concentrically laminated keratin riched horn cysts, keratoacanthoma which was similar as SCC while it was benign and could resolve spontaneously (5, 6, 10, 16) yet can be differentially diagnosed based on their growth pattern and histopathology.

TLC has a benign clinical course, and there is no evidence of recurrence after complete excision (7, 8). Billingsley et al. (11) and Lai et al. (8) recommended Mohs micrographic surgery as the treatment to ensure complete surgical excision. Besides, full dose irradiation can also be applied to treat TLC and it is very important to offer careful follow-up to the patients.

In the early stage of our case, the clinical manifestations of TLC was not apparent. Our diagnosis was misleaded as BKC because of the eyelid margin only showed slight keratinization and roughness devoed of ulcers or bleeding. Conjunctival and corneal involvement was considered to be the sequel of BKC, and it affected both the meibomian gland and the ocular surface, leading to keratitis, corneal thinning or perforation, vascularization and scarring. BKC is a chronic inflammatory disorder induced by infections, immunologic conditions, dermatoses, toxic conditions, trauma and tumors (17). Rectifying the eyelid margin should be considered crucial in the treatment of BKC as they may mediate a chronic inflammatory reponse. The application of ointments and corticosteroids is effective to manage the BKC. However, the BKC caused by tumer or immunemediated diseases do not respond to therapy, particularly if it is resulted in conjunctival cicatricial changes or loss of eyelashes (18).

In our case, we excluded common causes of blepharositis, and did not comprehend that it was a rare tumor until the ulcer lesions appeared. Whats more, the penetrating keratoplasty and tumer complete excision were timely and effective.

## Data Availability Statement

The original contributions presented in the study are included in the article, further inquiries can be directed to the corresponding authors.

## Ethics Statement

This case report was approved by the Ethics Committee of Xiamen University affiliated Xiamen Eye Center and written informed consent was obtained from the patient. The patient consented to publish the image of her eye and other results of her medical examination in this manuscript or paper which may be published in this journal.

## Author Contributions

LZ designed this study, performed the literature search, and wrote the manuscript. ZL, HW, and SO designed this study. All authors have read and approved the manuscript.

## Funding

This study was supported by the National Natural Science Foundation of China [Grant Numbers: 82101084 and 82060173], China Postdoctoral Science Foundation [Grant Number: 2021M69898], the Xiamen Science and Technology Program for Public Wellbeing [Grant Number: 3502Z20209183], and Science and Technology Support Program of Guizhou Province [20204Y145]. The funders had no role in the study design, data collection and analysis, publishing decision, or preparation of the manuscript.

## Conflict of Interest

The authors declare that the research was conducted in the absence of any commercial or financial relationships that could be construed as a potential conflict of interest.

## Publisher's Note

All claims expressed in this article are solely those of the authors and do not necessarily represent those of their affiliated organizations, or those of the publisher, the editors and the reviewers. Any product that may be evaluated in this article, or claim that may be made by its manufacturer, is not guaranteed or endorsed by the publisher.

## Abbreviations

BKC: Blepharokeratoconjunctivitis
TLC: Trichilemmal carcinoma
BCC: Basal cell carcinoma
SCC: Squamous cell carcinoma
PTT: Proliferating trichilemmal tumors.
